# Sequence variants in malignant hyperthermia genes in Iceland: classification and actionable findings in a population database

**DOI:** 10.1038/s41431-021-00954-2

**Published:** 2021-08-31

**Authors:** Run Fridriksdottir, Arnar J. Jonsson, Brynjar O. Jensson, Kristinn O. Sverrisson, Gudny A. Arnadottir, Sigurbjorg J. Skarphedinsdottir, Hildigunnur Katrinardottir, Steinunn Snaebjornsdottir, Hakon Jonsson, Ogmundur Eiriksson, Gudjon R. Oskarsson, Asmundur Oddsson, Adalbjorg Jonasdottir, Aslaug Jonasdottir, Gisli H. Sigurdsson, Einar P. Indridason, Stefan B. Sigurdsson, Gyda Bjornsdottir, Jona Saemundsdottir, Olafur T. Magnusson, Hans T. Bjornsson, Unnur Thorsteinsdottir, Theodor S. Sigurdsson, Patrick Sulem, Martin I. Sigurdsson, Kari Stefansson

**Affiliations:** 1 deCODE Genetics/Amgen Inc., Reykjavik, Iceland; 2 Division of Anaesthesia and Intensive Care, Landspitali University Hospital, Reykjavik, Iceland; 3 Faculty of Medicine, University of Iceland, Reykjavik, Iceland; 4 School of Health Sciences, University of Akureyri, Akureyri, Iceland; 5 Department of Genetics and Molecular Medicine, Landspitali University Hospital, Reykjavik, Iceland; 6 McKusick-Nathans Institute of Genetic Medicine, Johns Hopkins University, Baltimore, MD USA

**Keywords:** Disease genetics, Genetics research

## Abstract

Malignant hyperthermia (MH) susceptibility is a rare life-threatening disorder that occurs upon exposure to a triggering agent. MH is commonly due to protein-altering variants in *RYR1* and *CACNA1S*. The American College of Medical Genetics and Genomics recommends that when pathogenic and likely pathogenic variants in *RYR1* and *CACNA1S* are incidentally found, they should be reported to the carriers. The detection of actionable variants allows the avoidance of exposure to triggering agents during anesthesia. First, we report a 10-year-old Icelandic proband with a suspected MH event, harboring a heterozygous missense variant NM_000540.2:c.6710G>A r.(6710g>a) p.(Cys2237Tyr) in the *RYR1* gene that is likely pathogenic. The variant is private to four individuals within a three-generation family and absent from 62,240 whole-genome sequenced (WGS) Icelanders. Haplotype sharing and WGS revealed that the variant occurred as a somatic mosaicism also present in germline of the proband’s paternal grandmother. Second, using a set of 62,240 Icelanders with WGS, we assessed the carrier frequency of actionable pathogenic and likely pathogenic variants in *RYR1* and *CACNA1S*. We observed 13 actionable variants in *RYR1*, based on ClinVar classifications, carried by 43 Icelanders, and no actionable variant in *CACNA1S*. One in 1450 Icelanders carries an actionable variant for MH. Extensive sequencing allows for better classification and precise dating of variants, and WGS of a large fraction of the population has led to incidental findings of actionable MH genotypes.

## Introduction

Malignant hyperthermia (MH) is a rare life-threatening pharmacogenetic disorder with the susceptibility inherited in an autosomal dominant pattern, but the disorder only happens upon exposure to a triggering agent (volatile anesthetics and succinylcholine). The “gold standard” for diagnosis of MH has been in vitro contracture test of muscle fibers in the presence of caffeine and halothane [1]. However, this test requires a surgical biopsy and is relatively complex to perform, so the conduct of these tests has been centralized to highly specialized laboratories. Currently, genetic testing may also be conducted to confirm diagnosis of MH susceptibility [2]. MH is most commonly due to protein-altering variants in the ryanodine receptor 1 (*RYR1*) gene, and less frequently in a gene producing calcium channel alpha-1s subunit (*CACNA1S*) [3]. Numerous sequence variants within those genes and others with a potential association with MH have been described, many of unknown significance. It is challenging to rule out MH susceptibility in individuals with pathogenic *RYR1* variants considering the incomplete penetrance of MH susceptibility, and individuals do not always exhibit symptoms following initial exposure to triggering agents. The revised European Malignant Hyperthermia Group guidelines recommend that an individual carrying a potentially MH-associated variant should be considered at risk of MH until contracture testing can be performed [2, 4]. Furthermore, *RYR1* and *CACNA1S* are listed among the 59 genes with recommendations to report incidental findings of medically actionable variants by the American College of Medical Genetics and Genomics (ACMG) [5]. In the last decade, the cost of whole-exome sequencing and whole-genome sequencing (WGS) has decreased allowing clinical application more feasible [6]. We and others have sequenced whole genomes or whole exomes of a large set of individuals to perform genetic studies of common and rare diseases [7–10]. These large-scale sequence datasets can be used as a reference of variant frequency in the context of clinical sequencing [11], but can also lead to incidental findings.

First, we describe a case of MH in an Icelander with unknown family history where a family-specific variant in *RYR1* was identified. We underline the value of extensive knowledge of sequence diversity in a population to precisely determine the age and frequency of the variant. Second, we take advantage of the WGS set of 62 thousand Icelanders, followed by imputation into a total of 166 thousand Icelanders (chip-genotyped and/or whole-genome sequenced individuals), to determine the carrier frequency of actionable pathogenic and likely pathogenic variants in *RYR1* and *CACNA1S*, according to current guidelines [12]. The detection of actionable variants causing MH susceptibility has the potential to alter the clinical management of patients harboring the variants by avoiding exposure to triggering agents during anesthesia.

## Materials and methods

### DNA whole-genome sequencing

Our whole-genome sequence dataset is a collection of DNA samples from 62,240 Icelanders who have participated in various disease projects at deCODE genetics. All participating individuals who donated blood or buccal samples signed informed consent. The identities of participants were encrypted using a third-party system approved and monitored by the Icelandic Data Protection Authority.

The methods used for WGS were as follows: paired-end libraries for sequencing were prepared from DNA samples (derived from blood or buccal swabs) using Illumina preparation kits (TruSeq DNA, TruSeq Nano, or TruSeq PCR-Free) according to the manufacturer’s instructions. Paired-end sequencing-by-synthesis was performed on Illumina sequencers (GAIIx, HiSeq 2000/2500, HiSeq X, or NovaSeq) to a target depth of 30×. Read lengths varied from 2 × 76 to 2 × 150 bp, depending on the instrument and/or sequencing kit used. Reads were aligned to the human genome assembly GRCh38 using the Burrows–Wheeler Aligner version 0.7.10 [13]. Alignments were merged into a single BAM file and marked for duplicates using Picard 1.117. Only non-duplicate reads were used for the downstream analyses.

### Variant calling and annotation

Variants were called using version 2014.4-2-g9ad6aa8 of the Genome Analysis Toolkit (GATK) [14], reads were called with GATK using a multi-sample configuration. The effects of sequence variants were annotated using release 80 of the Variant Effect Predictor (VEP-Ensembl) [15]. To be able to filter out genotypes over a certain frequency threshold we used allelic frequencies from phased genotypes of 32.5 million SNPs and INDELs from 28,075 Icelanders who have been whole-genome sequenced at deCODE genetics [10]. The p.(Cys2237Tyr) variant identified in the proband has been submitted to ClinVar (accession ID: SCV001623063). Other variants identified in this study have been deposited to the European Variation Archive (Project: PRJEB46486).

### Sanger sequencing

Internal Sanger sequencing was performed using BigDye^®^ Terminator chemistry on a 3730 system (Applied Biosystems, Thermo Fisher Scientific), with primers designed using the Primer 3 software.

### Haplotype sharing

Haplotype sharing was based on long-range phased genotypes in combination with Icelandic genealogy that allowed for parental origin of the alleles of interest to be determined [16, 17]. When dating the p.(Cys2237Tyr) variant in *RYR1*, we used these long-range haplotypes by identifying genotype discordance between relatives of the family of the proband.

Further details on our approach to WGS, genotyping, long-range phasing, and imputation have been described in previous publications [10, 18].

### Case description

A 10-year-old Icelandic boy, weighing 30 kg, was admitted for appendectomy. He had a previous history of an uneventful volatile anesthesia for tonsillectomy 5 years earlier. General anesthesia was induced with fentanyl and propofol, followed by succinylcholine and maintained with sevoflurane. A tracheal intubation was performed without complications although jaw muscle rigidity was noted. Following induction, a fever of 39.1 °C was detected in addition to hypercapnia with a peak pCO_2_ value of 80 mmHg (10.7 kPa) despite a minute ventilation of 267 mL/kg/min. A venous blood gas demonstrated a marked acidosis (pH 7.05), primarily due to respiratory acidosis (pCO_2_ 89 mmHg (11.9 kPa)) and lactic acidosis (Lactate: 4.40 mmol/L). A clinical diagnosis of MH was made. Maintenance anesthesia was switched to propofol infusion. The circuit filter and CO_2_ absorber were replaced and 2.5 mg/kg of dantrolene sodium was administered with reversal of symptoms. After completion of surgery, he was transferred to the intensive care unit (ICU) where he got supportive care and received additional dantrolene sodium for 24 h. Further laboratory testing demonstrated a profound increase in creatine kinase (115.1 μg/L, normal values: <7 μg/L) and myoglobin (41,615 μg/L, normal values: <76 μg/L) that normalized in a week. He was extubated the following day and discharged from the hospital on postoperative day 2 with full recovery. The proband had no overt signs of an overwhelming sepsis, he was afebrile and hemodynamically stable on presentation to both emergency room and operating theater, and had a normal white blood cell count and C-reactive protein. The parents of the proband provided consent for the publication.

## Results

### Genetic analysis

**Fig. 1:**
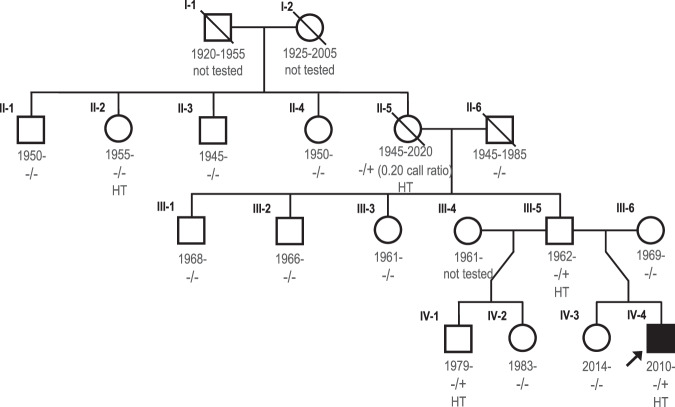
The pedigree of the proband (IV-4). HT corresponds to haplotype sharing of individuals encompassing the genomic coordinate of the variant in *RYR1* (chr19:38,496,455 [hg38]). [−/+] refers to confirmed carriers of the genotype, and [−/−] refers to non-carriers, confirmed by Sanger sequencing. Individual II-2 does not carry the variant; however, she shares the same haplotype background as individual II-5 (a confirmed carrier of the variant with 22 reads for reference allele and 6 for alternative allele). This implies that p.(Cys2237Tyr) led to mosaicism that occurred in the paternal grandmother (II-5) of proband. The years of birth and death of family members in generation I and II are 5-year rounded.

**Fig. 2:**
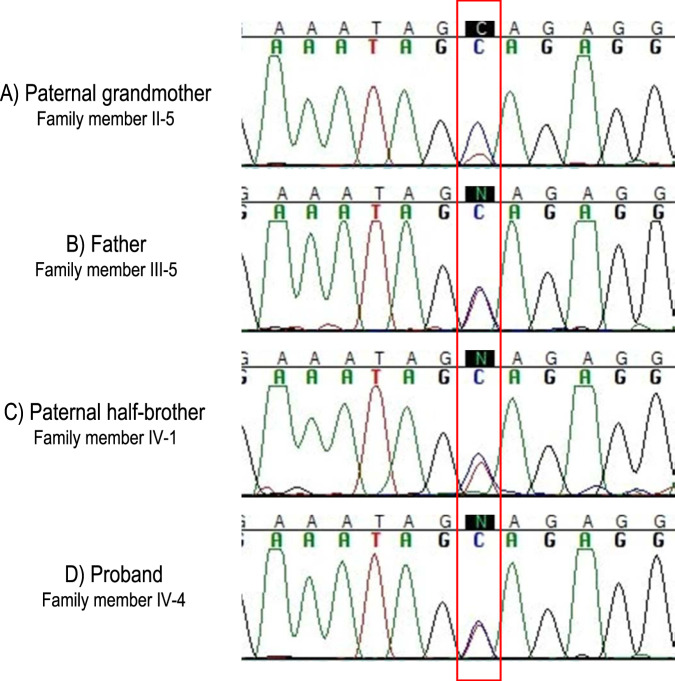
The Sanger sequencing chromatogram of the p.(Cys2237Tyr) variant at chr19:38,496,455 [hg38] in *RYR1* in all four carriers (highlighted with a red box). The reference allele is indicated with a blue peak and the alternative allele is indicated with a red peak. The variant is present in a heterozygous state in the proband (**D**), his paternal half-brother (**C**), and his father (**B**). The paternal grandmother (**A**) carries the variant in low allelic ratio, consistent with mosaicism, with ~0.20 allelic ratio.

DNA was extracted from peripheral blood from the proband with a diagnosis of MH (IV-4 in Fig. 1) and WGS was performed. This analysis yielded an ultra-rare, heterozygous missense variant in exon 41 of the *RYR1* gene (NM_000540.2:c.6710G>A p.(Cys2237Tyr); chr19:38,496,455 [hg38]). The majority of variants linked to MH in *RYR1* are clustered in three distinct regions (MH domain 1, 2, and 3) [19]. The p.(Cys2237Tyr) variant identified in the proband and his relatives is located within MH domain 2 that spans residues 2162–2458. The variant was detected in the proband and one additional first-degree relative out of 62 thousand WGS Icelanders. In the proband, we observed 18 sequencing reads for the reference allele and 19 reads for the alternative allele, resulting in a variant allelic ratio of 0.51. This genotype was confirmed with Sanger sequencing (Fig. 2D).

### Determining the age and origin of p.(Cys2237Tyr)

Sanger sequencing samples from 14 close relatives of the proband uncovered the p.(Cys2237Tyr) variant in three additional family members (Figs. 1 and 2A–C). Samples from 14 close relatives of the proband were Sanger sequenced (Fig. 1). This uncovered the p.(Cys2237Tyr) variant in three additional family members (Fig. 2A–C). The variant is present in the proband’s father (III-5), paternal half-sibling (IV-1), and paternal grandmother (II-5). Neither the proband’s father (III-5) nor half-sibling (IV-1) had undergone general anesthesia, and no history of a potential anesthetic reaction in the paternal grandmother (II-5) was available.

The paternal grandmother (II-5) harbors the variant with a low allelic ratio on WGS, 0.21 (6 out of 28 WGS reads), and 0.20 allelic ratio on Sanger sequencing (Fig. 2A). This is consistent with mosaicism in the paternal grandmother of the proband (II-5), as the allelic ratio deviates from the expected heterozygous allelic balance of 50/50 (*p* value = 0.0015). However, in the three descendants of the paternal grandmother (II-5) carrying the variant (III-5, II-1, and IV-4 the proband), the allelic ratio does not deviate from the expected allelic balance of 50/50 ratio. This suggests that the variant was transmitted from the paternal grandmother (II-5) to her descendants and is present in mosaic form in her somatic and germline cells. Using haplotype sharing based on phased and parent-of-origin determined chip-genotypes (Illumina array) and WGS data, we found that all four confirmed carriers share a haplotype background (genomic coordinate chr19:3,890,643-53,628,382 [hg38]) containing the p.(Cys2237Tyr) variant. The haplotype containing the variant was traced to a maternally inherited haplotype in the proband’s paternal grandmother (II-5). The same haplotype is also observed in her younger sister (II-2), who is not a carrier of the p.(Cys2237Tyr) variant. Taken together, the allelic imbalance in the paternal grandmother of the proband (II-5) and the absence of the identified variant in her sister (II-2), despite a shared maternal haplotype encompassing *RYR1*, is consistent with an early de novo mutation in the II-5.

Overall, the p.(Cys2237Tyr) variant is found in four individuals within three generations in the proband’s family (Fig. 1). No additional carriers of this variant were found in WGS data from over 62 thousand Icelandic individuals (a population of 360 thousand) or in WGS data from 30 thousand foreign individuals (mainly from European countries) sequenced at deCODE genetics. This suggests that the p.(Cys2237Tyr) variant observed in Iceland is a de novo mutation that took place in the paternal grandmother when she was an early embryo in a cell with descendants that contributed to both somatic and germ cells. The p.(Cys2237Tyr) variant has been reported before outside of Iceland in a single MH family in the Netherlands. Diagnosis of the index patient of that family was confirmed with a positive in vitro contracture test [1].

### Actionable MH variants in the Icelandic population

The ACMG guidelines recommend reporting of incidental findings in *RYR1* and *CACNA1S* of previously classified pathogenic and likely pathogenic variants in the context of MH [5]. By October 2020, a total of 2728 coding or splice site variants in *RYR1* are represented in ClinVar of which 367 are considered to be actionable since they are classified as pathogenic and/or likely pathogenic, after excluding variants with conflicting interpretation. The annotation in ClinVar database is not specific for a given phenotype; however, individuals with pathogenic or likely pathogenic variants linked to *RYR1*-associated myopathies are reported to be at risk for MH during general anesthesia and should avoid triggering anesthetics [4, 20]. In a set of 62 thousand WGS Icelanders, we detected 369 coding or splice variants in *RYR1* with minor allele frequency (MAF) <0.1% (Supplementary Table 1). Out of these 369 variants (MAF < 0.1%), we observed 13 actionable variants carried by 43 out of 62 thousand WGS Icelanders. This corresponds to a carrier frequency of 1 in 1450 of actionable *RYR1* variant, or 0.069% (out of all WGS Icelanders). Through imputation (166K chip-genotyped Icelanders) and Sanger sequencing of relatives, we identified 39 additional carriers of actionable *RYR1* variants, bringing the total to 82 carriers. This results in 1 in every 2000 individuals (chip-genotyped Icelanders) carrying a known pathogenic variant in *RYR1* (Tables 1 and 2). For *CACNA1S*, a total of 764 coding or splice site variants are represented in ClinVar, of which 36 variants are considered to be actionable. We detected 125 coding or splice site variants in *CACNA1S* with MAF < 0.1% out of 62K WGS Icelanders (Supplementary Table 2), but none are actionable (Table 1).

**Table 1:** Coding and splice variants (MAF < 0.1%) in *RYR1* and *CACNA1S* detected in the Icelandic population.

Gene	Carriers of any variant out of 62K WGS (no. of variants)	Carriers of P/LP variants out of 62K WGS (no. of variants)	Imputed^a^ carriers of P/LP variants out of 166K (no. of variants)
*RYR1*	2751 (369)	43 (13)	82 (13)
*CACNA1S*	799 (125)	0 (0)	0 (0)

*P/LP* pathogenic and likely pathogenic variants on ClinVar.

^a^Chip-genotyped and/or whole-genome sequenced individuals.

**Table 2:** Actionable variants in *RYR1* detected in the Icelandic population (MAF < 0.1%) that have previously been classified likely pathogenic or pathogenic in ClinVar.

	Chromosomal position [hg38]	Carriers out of 166K at deCODE genetics	MAF	HGVSc NM_000540.2	HGVSp NP_000531.2	Variant type	ClinVar interpretation (no. of submissions)	MH diagnosis	Reported phenotype for variant on ClinVar
	chr19:38455328	1	–	c.1534G>A	p.(Glu512Lys)	Missense	Pathogenic (1)	Unknown	CCD
	chr19:38464723	48	0.015%	c.2870+1G>A	.	Splice donor	Pathogenic (1) Likely pathogenic (1)	Unknown	RYR1-related disorders
	chr19:38469433	1	–	c.3686_3699del	p.(Met1229fs)	Frameshift	Pathogenic (1)	Unknown	RYR1-related disorders
	chr19:38485838	3	–	c.5183C>T	p.(Ser1728Phe)	Missense	Pathogenic (2) Likely pathogenic (2)	Unknown	MH
	chr19:38496466	4	–	c.6721C>T	p.(Arg2241Ter)	Stop gained	Pathogenic (10) Likely benign (1)	Unknown	Minicore myopathy, CCD and MH
	chr19:38499644	1	–	c.7039_7041GAG	p.(Glu2348del)	Inframe deletion	Drug response pathogenic (2)	Unknown	MH^a^
	chr19:38500643	3	–	c.7361G>A	p.(Arg2454His)	Missense	Drug response pathogenic (5)	Yes	MH^a^
	chr19:38525455	8	0.0028%	c.10579C>T	p.(Pro3527Ser)	Missense	Pathogenic (1)	Unknown	CCD
	chr19:38527645	4	–	c.10687-2A>G	.	Splice acceptor	Likely pathogenic (1)	Unknown	RYR1-related disorders
	chr19:38537933	1	–	c.11662C>T	p.(Gln3888Ter)	Stop gained	Pathogenic (1)	Unknown	RYR1-related disorders
	chr19:38572182	1	–	c.13910C>T	p.(Thr4637Ile)	Missense	Pathogenic (2)	Unknown	CCD
	chr19:38573288	6	0.0018%	c.14110C>T	p.(Arg4704Ter)	Stop gained	Pathogenic (1)	Unknown	RYR1-related disorders
	chr19:38580439	1	–	c.14581C>T	p.(Arg4861Cys)	Missense	Pathogenic (5)	Unknown	CCD and MH
Total	13 variants	82 carriers							

*MH* malignant hyperthermia, *CCD* central core disease, *CNM* centronuclear myopathy.

^a^Diagnostic mutation for MH according to EMHG.

## Discussion

First, we present a case of MH in a 10-year-old boy harboring a family-specific variant in *RYR1*, p.(Cys2237Tyr), which is classified as likely pathogenic. Second, we estimate overall carrier frequency of actionable variants in *RYR1* and *CACNA1S* among 62K WGS Icelanders, finding that 1 in every 1450 WGS Icelanders carries an actionable *RYR1* variant and none carry actionable *CACNA1S* variants.

The presentation of MH in the proband, a 10-year-old boy receiving an inhaled anesthetic who additionally received succinylcholine, was classic. It should be noted that while additional dantrolene was given in the ICU, it is not required after normalization of PaCO_2_ and with a decreased core temperature, although monitoring is required [21]. When the clinical status of the proband was applied to the clinical grading scale to predict MH susceptibility, the proband received a score of 70 pts (15 pts each for masseter spasm, CK elevation, respiratory acidosis, inappropriate temperature increase, and 10 pts for acidosis), rendering the likelihood of an MH episode as almost certain [22]. According to the European Malignant Hyperthermia Group, the suggested diagnostic pathway for patients with MH suspicion includes either genetic testing or in vitro contracture testing [2], which has largely been centralized to increase diagnostic accuracy. A genetic workup was performed identifying a *RYR1* variant (p.(Cys2237Tyr), chr19:38,496,455 [hg38]). The p.(Cys2237Tyr) variant is rare, absent from 212 thousand individuals in gnomAD reference database (v2.1 and v3.0) [11]. This same variant was reported positive for in vitro contracture testing in a case [1], and pathogenic prediction tools identify a high risk of damaging effect to the protein function [23]. Therefore, the variant classifies as likely pathogenic based on both MHS/RYR1-specific adaptation of the ACMG criteria, and the genetic scoring matrix from The European Malignant Hyperthermia group [24, 25]. This variant is currently not listed in ClinVar, a genotype–phenotype relationship reference database. The frequency of a sequence variant is a key determinant in the classification of pathogenicity, but it can be difficult to assess precisely. In Iceland, the extent of WGS and genotyping of 20% and 50% of the population, respectively, allows us to precisely determine the allelic frequency, the background haplotype, and the generation where the mutation that led to the variant happened. The p.(Cys2237Tyr) mutation led to mosaicism present in both germline cells of the proband’s paternal grandmother and her somatic cells (II-5). This variant was then inherited by three of her descendants, including the MH proband. Previously, this variant was reported once in a single individual outside of Iceland with an episode of MH, indicating that the variant identified in the Icelandic proband corresponds to a recurrent mutation. We note that the variant is located in MH domain 2, a region enriched for pathogenic missense variants between residues 2162 and 2458. The genetic workup identified two other alive family members without an anesthetic exposure who are likely to be susceptible to MH, out of 14 tested family members [2]. Given the large size of the *RYR1* gene (5038 amino acids, and ranks 27th largest out of 19,689 genes) and multiple variants within the gene that are of uncertain clinical significance, it is of utmost importance to attempt a molecular diagnosis of MH cases, and to distribute information about pathogenic variants.

Based on a large set of WGS Icelanders (*n* = 62 thousand), we determined that 1 in 1450 individuals carries one of the 13 actionable variants likely to cause MH in Iceland, all within *RYR1* (Table 2). After complementing WGS by imputation into a larger set followed by Sanger sequencing, we identified a total of 82 individuals carrying actionable variants likely to cause MH susceptibility.

In summary, this report shows how extensive knowledge of sequence diversity is instrumental in two different contexts for one condition. First, on an individual level, this allows interpretation of clinical sequencing results where the extent of the sequencing combined with the genealogy allows for precise dating of variants and assessing their pathogenicity. Second, at a population level, the detection of actionable variants in a large dataset has the potential to assist in the delivery of healthcare.

## Supplementary information

Supplementary Table 1

Supplementary Table 2

## Data Availability

The datasets supporting the conclusions of this article are included within the article (and its additional files).
